# Costs of providing food assistance to HIV/AIDS patients in Sofala province, Mozambique: a retrospective analysis

**DOI:** 10.1186/1478-7547-11-20

**Published:** 2013-08-28

**Authors:** Mariana Posse, Rob Baltussen

**Affiliations:** 1 Faculty of Economics and Management, Catholic University of Mozambique, Beira, Mozambique; 2 Department of Primary and Community Care, Radboud University Nijmegen Medical Centre, P.O Box 9101, 6500 HB, Nijmegen ELG 117, the Netherlands

**Keywords:** ART, HIV/AIDS, Food assistance, Costs

## Abstract

**Background:**

As care and antiretroviral treatment (ART) for people living with HIV/AIDS become widely available, the number of people accessing these resources also increases. Despite this exceptional progress, the estimated coverage in low- and middle-income countries is still less than half of all people who need treatment. In addition, treatment discontinuation and non-adherence are still concerns for ART programs. Governments and partner institutions have sought to implement a variety of interventions addressing the main reasons behind the low coverage of, discontinuation of, and non-adherence to ART. Food assistance is one of those interventions; increasing evidence suggests that this type of intervention has the potential to improve ART outcomes. However, to our knowledge, no study has estimated its costs in detail. The objective of this study was to assess the costs of a program providing food assistance to HIV/AIDS patients in Sofala province, Mozambique, in 2009.

**Methods:**

We performed a retrospective analysis of the costs of providing food assistance, based on financial and economic costs. We used the ingredients approach to estimate costs, which involved multiplying the total estimated quantities of goods and services actually employed in providing the intervention by their respective unit prices.

**Results:**

In 2009, the cost of providing food assistance to HIV/AIDS patients was $2.27 million, with capital and recurrent costs accounting for 1% and 99% of total costs, respectively. Food made up the largest component, at 49% of total costs. At 24%, transport operating costs were the second largest item. The cost per patient served was $288 over 3 months.

**Conclusion:**

The food distribution program carries significant costs. To assess whether it provides value for money, the present study results should be interpreted in conjunction with the program’s impact, and in comparison with other programs that aim to improve adherence to ART. Our costing analysis revealed important management information, indicating that the program incurred relatively large overhead costs. This result raises questions regarding the efficiency of implementing this food distribution program.

## Background

As care and antiretroviral treatment (ART) for people living with HIV/AIDS becomes widely available, the number of people accessing them also increases. At the end of 2010, 6.65 million people were receiving treatment in low- and middle-income countries, an increase of 27% from December 2009 [1]. Despite this exceptional progress, the estimated coverage in low- and middle-income countries is still less than half of all people who need treatment [1]. In addition, treatment discontinuation and non-adherence are still concerns for ART programs [2,3], since they may cause drug resistance [3-5], virological failure [6], accelerated disease progression [7], increased hospitalizations, and, consequently, increased health care costs [8].

Governments and partner institutions such as the World Health Organization (WHO), the Joint United Nations Program on HIV/AIDS, the United Nations World Food Program (WFP), and major HIV/AIDS initiatives such as the US President’s Emergency Plan for AIDS Relief have sought to implement a variety of interventions addressing the main reasons behind the low coverage of, discontinuation of, and non-adherence to ART. Food assistance, which is aimed at improving the physical ability of patients to take ART and to render it more clinically effective, is one of those interventions [9-11]. There is increasing evidence that this type of intervention has the potential to increase household consumption expenditure, to improve food security, nutritional status, and pre-and post-adherence to ART, and to delay disease progression [12-19]. However, to our knowledge, no study has estimated its costs in detail.

Estimating program costs is important for a number of reasons. First, costs could be used to assess allocative efficiency by determining whether a program provides value for money, for example, by comparing its costs and effects to those of other investments in health (e.g. food assistance versus tuberculosis control). Second, costs could be used to assess technical efficiency by determining whether a program provides value for money within the same domain, for instance, by comparing its costs and effects with those of alternative options for providing food assistance. Third, it could be used for management purposes, such as planning improvement by identifying larger-cost items and potentials for savings [20,21]. In this study, we assessed the program costs of providing food assistance for HIV/AIDS patients in the Sofala province of Mozambique in 2009.

## Methods

### Description of the food assistance program

We evaluated the food assistance program as implemented by the United Nations WFP in collaboration with Health Alliance International (HAI) and the Ministry of Health. HAI is a non-governmental organization that initiated operations in Mozambique in 1987. Its mission is to support the development of policies that foster social and economic equity for all, with a focus on public-sector health systems and a progressive realization of the right to health. In 2003, in partnership with the government of Mozambique, HAI began to support the implementation and expansion of ART for HIV/AIDS patients. It is within this partnership that HAI also collaborates with the WFP in providing food assistance for HIV/AIDS patients in Sofala province.

The WFP is responsible for the provision of food, health centers are responsible for the identification of eligible patients, and HAI is responsible for the distribution of food to patients. This intervention is provided to HIV/AIDS patients of all ages, including children. It is also provided to tuberculosis patients and to pregnant and breast-feeding women enrolled in prevention of mother-to-child transmission programs. The objective is to ensure patient nutritional recovery and treatment success.

The criteria for inclusion in the food assistance program were body mass index below 18.5 kg/m^2^_,_earning no income or less income than the monthly national minimum income, and many dependents. Patients were identified before or after they started ART. They were first identified by a social worker; patient eligibility was later confirmed by a clinician. After being identified, patients received an identification card that entitled them to collect food at the distribution site, usually a warehouse or health facility. They received food assistance once per month for a period of three months, after which they were reassessed to determine whether their nutritional status had improved. If improved, patients were discharged from the program; otherwise, the patients remained in the program for an additional 3–6 months. Food assistance consisted of 10 kg of soya, 5 kg of cowpeas, and 25 kg of maize.

This study was approved by the Mozambican National Bioethics Committee.

### Calculation of costs

We undertook the costing analysis from a provider perspective: the costs incurred by the three organizations implementing the food assistance program (HAI, WFP, and health facilities). The cost calculations apply to all 13 districts in Sofala province over the implementation period of 2009.

To estimate costs incurred by HAI and the health facilities, we used the ingredients approach by multiplying the total estimated quantities of goods and services actually employed in providing the intervention by their respective unit prices. This approach is useful because it makes it possible to later identify what is driving the total costs, and how to improve the use of available resources [22]. We obtained the details of the financial costs from the government expenditure records and from HAI’s administrative records.

To estimate the costs incurred by WFP at the program level in Sofala province, we used a top-down approach based on the number of people reached by the intervention; we allocated transport and other program costs together (rather than separating transport and program) because we did not have information on the breakdown of costs at the national level by line item. We compared the total number of people served at the national level with the number of HIV/AIDS patients served in Sofala province, and used this proportion to estimate the costs at the province level. We distributed the program costs among categories according to the percentage of each category out of the total value of the national budget. For example, if staff costs corresponded to 6% of total value in the WFP budget allocated to Mozambique, we also allocated 6% of the food program costs in Sofala province for staff costs. We obtained details on costs incurred by the WFP in their budget allocated to Mozambique [23].

### Identification of costs

To determine the costs of the intervention, we interviewed people in various job categories and visited five places where the intervention was actually implemented. These places were selected based on convenience of access. We divided the costs identified according to the activities performed in two phases of program implementation of the program: the start-up and post start-up phases. For the start-up phase (the time between the decision to implement an intervention and starting its delivery to the first beneficiary), we only included data on the costs of initial training (as a capital cost). For the post start-up phase (the period of implementation), we included costs based on three main activities: planning and monitoring, patient identification, and food distribution.

For planning and monitoring, we only included staff costs. For the health facilities, we based our estimates on expenditure data and included staff costs at the provincial level (health director, chief medical doctor, and nutritional assistant). For HAI (the organization responsible for food distribution), we included staff costs at the national and provincial levels (program manager, program coordinator, and nutritional assistant) and at the district level (multidisciplinary team manager and administrative assistant). To assess staff time spent in these activities, we interviewed four administrative assistants, one nutritional assistant, the program manager, the program coordinator, and three multidisciplinary team managers. The nutritional assistant provided us with estimates of time spent by the health director and the chief medical doctor.

Similarly, for patient identification, we only included staff costs incurred at the district level by health facilities, since these were responsible for the identification of patients eligible to receive food assistance. We included the costs of 13 health facilities, one health facility per district. To assess staff time spent at the health facilities, we interviewed five social workers, who also provided estimates of time spent by medical doctors.

Regarding food distribution, we only included the costs incurred by HAI, since it was responsible for food distribution. We included the costs of transport, materials and supplies, equipment, training, and staff at the district level (Table 1). To assess staff time, we interviewed three warehouse managers, five distribution members, and three guards.

**Table 1:** Costs included in the analysis

	**Description of costs**
**Capital**	Training: initial and ongoing
	Equipment: computer, desks, chairs, pallets and measuring devices
**Recurrent**	Staff
	*National level*:program managers
	*Provincial level*: health provincial director, chief medical officer, nutritional assistant, multidisciplinary team manager, and administrative assistant
	*District level*: medical doctor, social assistant, warehouse manager, food distribution members, and guards
	Food: maize, soya, and cowpeas
	Materials and supplies: cards, cleaning material, office supplies, and other consumables
	Transport operational costs: fuel, depreciation, vehicle running costs, and vehicle maintenance
	Other recurrent costs: car rental, warehouse and office rental, product storage and handling and staff duty travel and communication

For WFP, the organization that provided the food, we included the costs of staff, transport, and equipment. We obtained details about the price and quantities of food from an interview with the head of the program unit in Sofala province.

### Valuation of costs

We measured financial costs at the time the expenditure was incurred. We included staff, food, materials and supplies, and transport operational costs as recurrent costs (Table 1). Foreign exchange transactions were expressed in the local currency by applying the average market exchange rate for 2009 (27.28 meticais = $1). We used the monthly income for each job category to estimate staff costs incurred by HAI and health facilities. We multiplied the time spent in the intervention by the monthly income and multiplied the result of that product by 12 months. For example, at HAI there were two nutritional assistants who spent 100% of their time in the intervention and received $1077 as their monthly income. Therefore, we performed the following calculation: (2 × 1 × $1077) × 12 = $25, 848.

We calculated economic costs by annuitizing capital goods on the basis of their useful life at a discount rate of 3%, as recommended by most guidelines [22]. We included initial and ongoing training and equipment as capital costs (Table 1). We annuitized initial training over a period of 10 years and ongoing training over a period of one year. We valued equipment using the WHO-CHOICE estimates on price (2009 international dollars) [24]. We used WHO-CHOICE equipment lists because there was no information available about the purchase prices of equipment used in the intervention. We valued office space by using rental prices, which also reflect opportunity costs. We did not include the overhead costs (office equipment, furniture, electricity, and water) of HAI and health facilities.

We calculated the cost per patient served by dividing the total costs of providing food assistance by the number of patients served (7,882). We implemented a sensitivity analysis to determine whether the results were sensitive to discount rate, staff time use, equipment prices, and the overhead costs of HAI and the health facilities.

## Results

For the year 2009, the costs of providing food assistance to HIV/AIDS patients was $2,271,656 (Table 2), with capital and recurrent costs accounting for 1% and 99% of the total cost, respectively. Food made up the largest component (48.7% of total cost), and transport operating costs were the second largest item (23.5%). The cost per patient served was $288. 21 over 3 months.

**Table 2:** Program costs ($USD) in 2009

	**Cost per item**	**% of total costs**	**Cost per person**
**Capital**			
Initial training	463	0.0	0.06
Ongoing training	3,876	0.2	0.49
Equipment	11,453	0.5	1.45
**Total capital costs**	**15,792**	**0.7**	
**Recurrent**			
Food	1,105,518	48.7	140.26
Staff	335,993	14.8	42.63
Materials and supplies	7,983	0.4	1.01
Transport operating costs	534,292	23.5	67.79
Other recurrent costs	272,079	12.0	34.52
**Total recurrent costs**	2,255,864	**99.3**	**288.21**
**Total program costs**	**2,271,656**		

**Figure 1:**
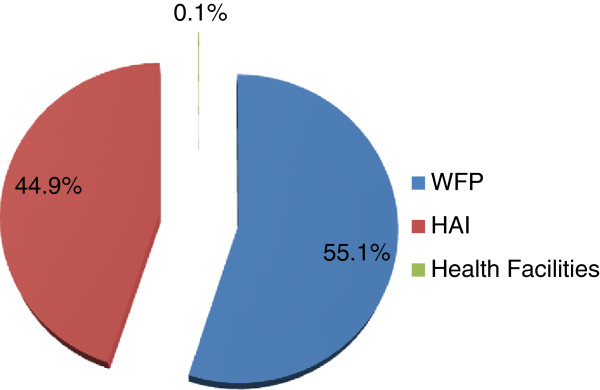
Contribution to the food assistance program by implementing organization.

When the total costs of providing food assistance were disaggregated for each partner, we determined that WFP incurred most of the costs (55.1%), followed by HAI (44.9%) and health facilities (0.1%; Figure 1). When the costs were broken down by activity, we observed that food distribution incurred most of the costs (36.7%), followed by planning and monitoring (14.6%). Patient selection was associated with the lowest cost (0.1%; Table 3).

**Table 3:** Contribution to the food assistance program by activity ($USD)

**Activity**	**Health facilities**	**HAI**	**WFP**	**Sub-total**	**% of total costs**
Monitoring/planning	49.90	185741.36	145905.4	331,696.66	14.6
Patient selection	1187.01	0.00	0.00	1187.01	0.1
Food distribution	0.00	833254.26	0.00	833254.26	36.7
Food	0.00	0.00	1105517.88	1105517.88	48.7
**Total**	1,236.91	1,018,995.62	1,251,423.28	2,271,655.81	100

**Figure 2:**
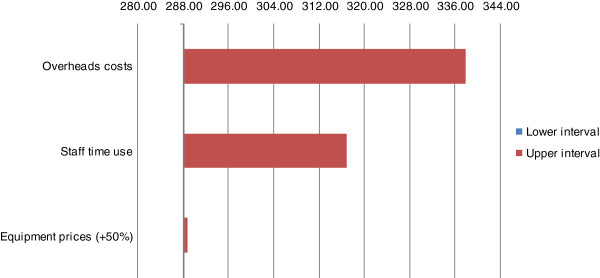
Sensitivity analysis (costs are given in $USD).

We used sensitivity analyses to assess whether our results were sensitive to discount rate, staff time use, equipment prices, and overhead at HAI and the health facilities. When the discount rate was increased to 5% and 10%, the cost per patient increased to $288.25 and $288.36, respectively (not reported in Figure 1). When 5% of the total cost of providing food assistance was included in the analysis as HAI and health-facilities’ overhead costs, the cost per patient increased to $317.00. When the time used by staff was increased from the minimum of 2.5% to 100%, the costs per patient increased to $338.00. When equipment prices were increased by 50%, the cost per patient increased to $288.83. Therefore, our results were not sensitive to changes in the discount rate, but were sensitive to overhead costs and to staff time use (Figure 2).

## Discussion

This paper provides the first detailed cost estimation of food assistance to HIV/AIDS patients in Mozambique. Our results indicate that the cost of providing food assistance over three months was $288 per HIV/AIDS patient in 2009. Recently, the annual cost for a patient on first-line ART in Mozambique was estimated at $294 [25]; the addition of a food assistance program to ART delivery would double these costs. It is difficult to assess whether integrating food assistance with ART delivery is the optimal strategy to reduce food insecurity, and two important considerations play a role in this assessment.

First, we previously reported the results of a retrospective impact analysis that suggested that the program has not significantly impacted adherence to ART [26]. However, other studies in low-income countries showed that integrating food assistance with ART may improve ART outcomes [12-19]. A possible explanation for the difference in findings includes the use of other outcome measures, since we only evaluated the impact on treatment adherence; food assistance can also directly affect ART outcomes in the sense that well-nourished patients are more responsive to treatment. These findings on the impact of food assistance should thus be interpreted with caution.

Second, it is important to note that food assistance can be justifiable from a social protection perspective, since it fulfills four main functions: (i) to ensure the minimum acceptable consumption levels of people who are already in difficulty, (ii) to prevent people who are susceptible to adverse events and shocks from becoming more vulnerable (by stopping them from having to sell their assets), (iii) to promote people’s ability to become less vulnerable in the future (by helping them to build assets and achieve stronger livelihoods), and (iv) to promote social justice through promoting the rights and empowerment of the poor and vulnerable [27].

Our findings reveal important information for program budgeting and for the identification of potential cost savings. Notably, the costs of food distribution were as high as the costs of the food itself, both accounting for approximately half of the total costs. If the food program were scaled up, little economies of scale should be expected, as these costs are all variable.

Several of the limitations of the present study may be addressable by future investigations. First, we only included data on the costs of training for the start-up phase, which may have underestimated the true costs incurred during this phase. Second, we did not include the overhead costs of the HAI and the health facilities in our analysis, but these costs may be significant. Third, we did not include the time and travel costs that patients incur to receive food assistance. Insights into these patient costs would allow program decisions regarding the optimal location for food distribution. Fourth, we used a top-down approach to estimate costs incurred by the WFP. This allocation of program costs on the basis of the number of people reached may have contributed to the high share of the WFP in total costs. Future research could attempt to address this limitation by measuring all resource use via a more detailed approach, such as the ingredients approach, in order to improve cost estimation. Fifth, we only analyzed the costs of the food and of program implementation, and did not consider the quality of the implementation. Future research could address this issue by including an assessment of the process of beneficiary targeting. Insights into these issues would allow program managers to better understand whether the program was delivered as expected and perhaps aid in explaining why a program is or not effective.

Despite calls for more economic analysis of interventions to improve treatment outcomes [17,28-31], to our knowledge this study is the first to provide a detailed cost estimation of food assistance to HIV/AIDS patients. We have attempted to capture, as fully as possible, all costs associated with its implementation.

## Conclusions

The food distribution program carries significant costs. To assess whether it provides value for money, the present study results should be interpreted in conjunction with the program’s impact, and in comparison with other programs that aim to improve adherence to ART. Our costing analysis revealed important management information, in the sense that the program incurred relatively large overhead costs. This observation raises questions regarding the efficiency of its implementation.

## Competing interests

The authors declare that there are no competing interests.

## Authors’ contributions

Both authors contributed to the design of the study. MP collected and analyzed data and drafted the manuscript. RB contributed advice on all aspects of study implementation and a critical revision of data analysis, interpretation of the results, and the manuscript. Both authors approved the manuscript.
